# Immunomagnetic isolation and in vitro expansion of human uveal melanoma cell lines

**Published:** 2008-01-10

**Authors:** Jonathan J. Cools-Lartigue, Cristin S. McCauley, Jean-Claude A. Marshall, Sebastian Di Cesare, Francois Gregoire, Emilia Antecka, Patrick Logan, Miguel N. Burnier

**Affiliations:** The Henry C. Witelson Ophthalmic Pathology Laboratory and Registry, McGill University Health Center, Montreal, PQ, Canada

## Abstract

**Purpose:**

Uveal melanoma (UM) is the most common intra-ocular tumor in adults. Despite advances in diagnosis and treatment, the survival rate of UM has not increased in the last several decades. Approximately 50% of patients will die as a consequence of metastatic disease with the majority of metastases localized to the liver. Due to the lack of lymphatics in the eye, hematogenous dissemination is the predominant means by which UM cells escape the primary site. Our laboratory has recently demonstrated the presence of circulating malignant cells (CMCs) in the blood using both animal models and clinical trails involving UM patients. Current data suggests that all UM patients will be positive for CMCs after diagnosis. Furthermore, some of the phenotypic changes that are necessary for metastatic growth may occur while the cells are circulating in the blood. In this study, we evaluated the efficiency of a panel of antibodies to immunomagnetically isolate CMCs for the purpose of in vitro expansion and genetic, immunological, and phenotypic characterization.

**Methods:**

In this study, five human uveal melanoma cell lines (92.1, MKT-BR, OCM-1, SP6.5, and UW-1) were immunostained with a panel of antibodies against known melanoma cell surface markers. Staining with monoclonal antibodies PAL M2, NKI C3, NKI/Beteb, and 9.2.27 permitted the generation of a cell surface expression profile in these cell lines. The five human UM cell lines and 92.1 transfected with GFP were subsequently spiked into human blood at concentrations ranging from 1x10^6^ cells/ml to 10 cells/ml. Cells were immuno-magnetically isolated at concentrations as low as 10 cells/ml.

**Results:**

Immunomagnetic isolation of all five human UM cell lines tested at concentrations down to 10 cells/ml human blood was achieved only when antibodies were used in combination. Individually, the antibodies did not permit isolation of cells at physiologically relevant concentrations.

**Conclusions:**

The immunomagnetic isolation method presented in this study can be used to isolate CMCs at physiologically relevant concentrations and at sensitivities comparable to those seen in polymerase chain reactions (PCR). In addition, our data suggests that our method is more efficient and reliable for the isolation of CMCs in UM than the methods currently used.

## Introduction

Uveal melanoma is the most common intraocular tumor in adults and is associated with high mortality [1]. Over the past several decades, many advances have been made in terms of prognostic efficiency and treatment modalities, resulting in a reduction in patient morbidity [2]. Current prognostic methods rely on histopathological profiling of tumor sections derived from enucleations with prognostic markers including cell type, tumor size, and a mean of the 10 largest nucleoli [3]. More recently, prognosis has been inferred independently of traditional histopathological markers. For example, poor prognosis has been linked to chromosome 3 aberrations [4]. In addition, employment of radiation therapy in the treatment of uveal melanoma has largely replaced enucleation for smaller tumors thus sparing the orbit in many cases [2]. Despite these advances, mortality rates remain unchanged. Approximately 50% of uveal melanoma patients will die within 10 years from metastasis, which localizes predominantly to the liver [1,5].

Due to the lack of lymphatics in the eye, uveal melanoma spreads almost exclusively via hematogenous dissemination [5]. Current understanding of this neoplasm is based on information gathered from studies focusing either on primary tumors or their corresponding metastases. However, very little is known about tumor cells subsequent to their egress from the ocular environment and before their development in the liver. Malignant cells are thought to disseminate from the primary tumor early in tumorigenesis and remain in a clinically latent state until either the cells themselves or the host is receptive to the development of metastases [6]. The biologic activity of these circulating malignant cells (CMCs) remains unclear. However, evidence from this laboratory, which is based on microarray analysis of tumor cells derived from different a stages of the metastatic cascade in an animal model, has demonstrated distinct changes in gene expression as cells progress from the eye to the blood and to the liver [7]. Such evidence highlights the importance of further characterization and understanding of CMCs.

CMCs have been detected in several malignancies including breast cancer and cutaneous melanoma [8,9]. CMCs have also been detected in uveal melanoma by the polymerase chain reaction (PCR) in a clinical trial conducted at this laboratory [10]. While PCR is a valuable method for the detection of CMCs, it is not without its limitations. In addition to the variability associated with the sensitivity of CMC detection reported in the literature, it does not permit the genotypic or phenotypic characterization of malignant cells in the blood. The mere presence of CMCs in uveal melanoma patients does not appear to be of prognostic value. PCR identification of CMCs in uveal melanoma suggests that all patients become positive at some point during the disease progression despite the fact that only 50% will die. Thus, CMC positivity may not be an inherent predictor of the clinical outcome of uveal melanoma [10].

Immunomagnetic isolation of CMCs from uveal melanoma patients may circumvent the limitations associated with PCR. Such a technique could enable the isolation of viable neoplastic cells that could then be subjected to any downstream application. As a result, CMCs could be subjected to the same scrutiny currently reserved for primary tumors or their metastases such as the establishment of cell cultures and genotypic and phenotypic characterization. Such studies could potentially shed light on the clinical implications connected to CMC positivity in uveal melanoma. Immunomagnetic isolation of CMCs from solid tumors has been demonstrated in a variety of malignancies including colorectal cancer, breast carcinoma, osteosarcoma, cutaneous, and uveal melanoma [9,11-13]. However, the current application of this emerging technology in the characterization of uveal melanoma may be incomplete. Current CMC isolation relies on the use of a single antibody, which may result in an underestimation in the sensitivity of the technique. In our method, we examine both independently and in combination a panel of antibodies reported to bind antigens expressed on uveal melanoma cells for their ability to efficiently capture CMCs at physiologically relevant concentrations [14].

## Methods

### Cell culture

The human uveal melanoma cell lines 92.1, SP6.5, and MKT-BR were established by Dr. Jager (University Hospital Leiden, Leiden, The Netherlands), Dr. Pelletier (Laval University, Quebec, Canada), Dr. Belkhou (CJF INSERM, Strasbourg, France), respectively. Dr. Albert (University of Wisconsin-Madison, Madison, WI) established the OCM-1 and UW-1 cell lines [15,16].

The five human uveal melanoma cell lines were incubated at 37 °C in a humidified 5% CO_2_-enriched atmosphere. All cell lines were cultured in RPMI-1640 medium (Invitrogen, Burlington, Ontario, Canada), supplemented with 5% heat inactivated fetal bovine serum (FBS; Invitrogen), 1% fungizone (Invitrogen), and 1% penicillin-streptomycin (Invitrogen). Cells were fed bi-weekly, and the nonadherent 28SC cells were centrifuged at every feeding. 92.1, MKT-BR, OCM-1 SP6.5, and UW-1 were grown to confluence as a monolayer and passaged by treatment with 0.05% trypsin in EDTA (Fisher, Whitby, Ontario, Canada).

### Green fluorescent protein transfection

Uveal melanoma cell line 92.1 was transfected with green fluorescent protein (GFP) using lipofectamine as per the manufacturer's instructions (Promega, Invitrogen). Briefly, 2 μg of GFP was diluted in 100 μl OPTI-MEM serum-free medium. Cells were allowed to incubate overnight at 37 °C in a 5% CO_2_ environment. Cells were then grown in selective growth medium with 400 μg/ml G418 (Gibco, Ontario, Canada). Fluorescence was checked weekly using an inverted epi-fluorescent microscope (Nikon, Ontario, Canada).

### Cytospins

Cytospins were prepared from lines 92.1, MKT-BR, OCM-1, SP6.5, and UW-1 at a concentration of 250,000 cells/spin (Shandon Scientific, Cheshire, England). Cytospins were fixed in paraformaldehyde and stored at −20 °C until needed.

### Immunocytochemistry

Immunostaining was performed on cytospins of lines 92.1, MKT-BR, OCM-1, SP6.5, and UW-1 according to the avidin-biotin complex technique. Blocking of non-specific binding was performed by incubating samples in a Tris buffer containing 1% bovine serum albumin (TBS/1% BSA). Cytospins were incubated for 1 h at room temperature with one of the murine monoclonal antibodies NKI/Beteb (melanoma-associated antigen 100 kDa, 7 kDa), NKI/C3 (melanoma-associated antigen), or PAL-M2 (determinant discriminating nevo cellular nevi, malignant melanoma) diluted 1:100 as indicated by the manufacturer (Monosan, Uden, The Netherlands). Negative controls incubated without a primary antibody were included for each cytospin tested.

Immunostaining was performed on paraformaldehyde-fixed cytospins of lines 92.1, MKT-BR, OCM-1, SP6.5, and UW-1 cell lines using the primary antibody 9.2.27 (purified mouse anti-chondroitin sulfate monoclonal antibody; BD Biosciences PharMingen, Franklin Lakes, NJ) using the Envision system-AP (DakoCytomation, Carpinteria, CA). Negative controls incubated with mouse sera were included for each cytospin tested.

### Isolation of uveal melanoma cells from culture

Uveal melanoma cells were isolated using the CELLection Pan Mouse IgG Kit (Dynal Biotechnology, Oslo, Norway) consisting of 4.5 μm superparamagnetic polystyrene beads coated with monoclonal human anti-mouse IgG. Cells were isolated via the direct technique as described by the manufacturer. Uveal melanoma cell lines 92.1, MKT-BR, OCM-1, SP6.5, UW-1, and 92.1, transfected with GFP, were adjusted to a concentration of 1x10^6^ cells/ml in phosphate buffered saline (PBS) containing 0.1% BSA, and five 10-fold dilutions were performed to yield six cell concentrations ranging from 1x10^6^ to 10 cells/ml PBS/0.1%BSA. Cell suspensions from lines 92.1, OCM-1, and SP6.5 were added to CELLection Pan Mouse IgG Dynabeads pre-labeled with NKI/C3, NKI/Beteb, PAL-M2, or the combination of all three antibodies. Cell suspensions consisting of the remaining cell lines were added to CELLection Pan Mouse IgG Dynabeads pre-labeled with NKI/C3and NKI/Beteb in combination. Negative controls consisted of cells incubated with unlabeled beads. Cells were then eluted and plated in six-well plates containing 1 ml RPMI-1640 medium supplemented with 5% heat-inactivated FBS, 1% fungizone, and 1% penicillin-streptomycin. Cultures were monitored daily for growth via microscopic evaluation.

### Isolation of uveal melanoma cells from spiked human blood

Uveal melanoma cells were isolated via the direct technique using the CELLection Pan Mouse IgG Kit as described by the manufacturer. Human uveal melanoma cell lines 92.1, MKT-BR, OCM-1, SP6.5, UW-1, and 92.1 transfected with GFP were adjusted to a concentration of 1x10^6^ cells/ml PBS/0.1% BSA and five 10-fold serial dilutions were performed to yield six cell concentrations ranging form 1x10^6^ to 10 cells/ml of PBS/0.1% BSA. Cell suspensions were mixed with an equal volume of blood obtained from healthy volunteers. Mononuclear cells (MNCs) and CMCs were isolated using Ficoll Paque Plus. The washed, pelleted MNCs and CMCs were resuspended in PBS/0.1% BSA and incubated with CELLection Pan Mouse IgG Dynabeads pre-labeled with either a combination of monoclonal antibodies, NKI/C3 and NKI/Beteb, or 9.2.27. Negative controls consisted of cells incubated with unlabeled beads. Bound cells were eluted and plated in six-well plates containing 1 ml RPMI-1640 medium supplemented with 5% heat-inactivated FBS, 1% fungizone, and 1% penicillin-streptomycin. Cultures were monitored daily for growth via microscopic evaluation. All human blood samples for these experiments were obtained as per the IRB approved protocol.

## Results

### Immunocytochemical profile of the five human uveal melanoma cell lines

**Figure 1:**
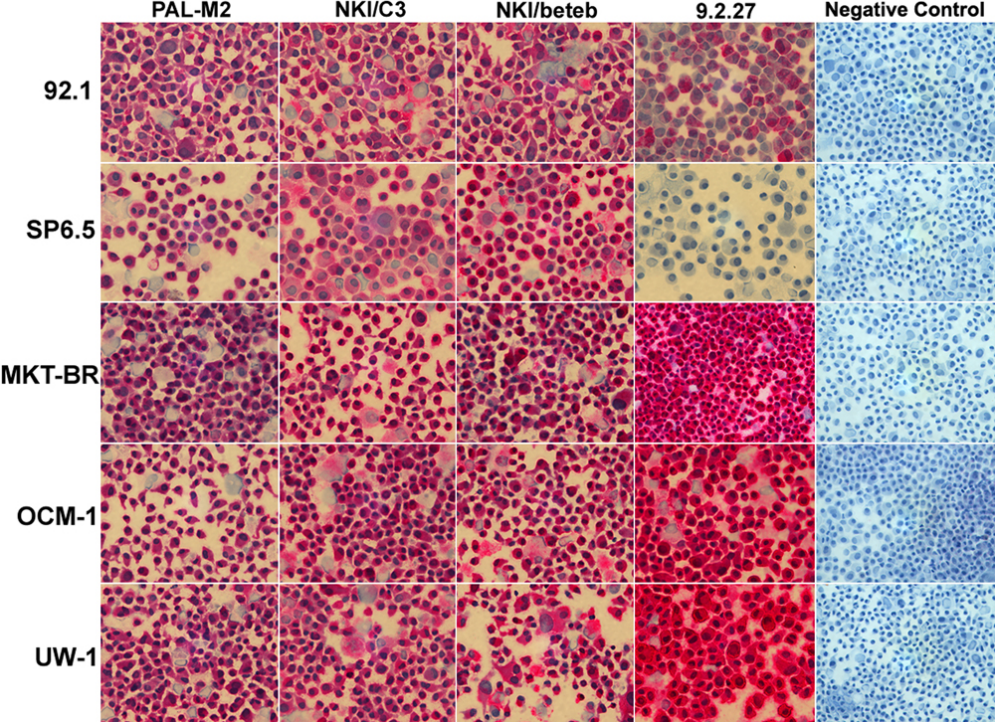
Immunohistochemical profile of 5 human uveal melanoma cell lines stained with monoclonal antibodies. Immunohistochemical profile of 5 human uveal melanoma cell lines (92.1, SP6.5, MKT-BR, OCM-1, and UW-1) stained with monoclonal antibodies PAL M2, NKI C3, NKI/Beteb, 9.2.27, and appropriate negative control. Each image is at 400x magnification.

The immunocytochemical staining profile for lines 92.1, MKT-BR, OCM-1, SP6.5, and UW-1 is depicted in Table 1. Immunostaining was graded subjectively as negative (-), low (+), moderate (++), and high (+++) as previously described by Makitie, Summanen et al. [17]. Three independent observers performed these evaluations based on the overall impression of the cytospins, which were examined under a light microscope at magnifications of 100x-400x. All cytospins stained positive using NKI/C3 with SP6.5 showing the lowest levels of expression (+; Figure 1). By contrast, all other cell lines exhibited moderate staining intensity (++) with this antibody (Figure 1). Staining patterns associated with NKI/Beteb were all moderate (++) except for UW-1, which was low (-). All cytospins demonstrated moderate levels of staining for PAL-M2 (++). Three cell lines displayed a high staining intensity (+++) with 9.2.27 with the exceptions of 92.1 and SP6.5. SP6.5 displated negative (-) levels of 9.2.27 expression as depicted in Figure 1. The 92.1 cell line displayed low levels (+) of staining intensity.

**Table 1:** Immunhistochemical staining profile for five human uveal melanoma cell lines.

	**Antibody/staining intensity**			
**Cell Line**	**PAL-M2**	**NKI-C3**	**NKI/beteb**	**9.2.27**
92.1	++	++	++	+
SP6.5	++	+	++	-
MKT-BR	++	++	++	+++
OCM-1	++	++	++	+++
UW-1	++	++	+	+++

Immunhistochemical staining profile for the five human uveal melanoma cell lines 92.1, MKT-BR, OCM-1, SP6.5, and UW-1 using monoclonal antibodies NKI/C3, NKI /Beteb, PAL-M2, and 9.2.2.7 as determined by three independent observers.

### Isolation of uveal melanoma cells from culture

Immunomagnetic isolation of uveal melanoma cells from culture was used to validate our method and determine its sensitivity in our uveal melanoma cell lines. The three uveal melanoma cell lines used to initially validate our method were 92.1, OCM-1, and SP6.5 because of their observed immunocytochemical profile. Initially, CELLection Dynabeads were pre-labeled with NKI/C3 only. This permitted the capture of cells at concentrations of 1x10^6^ to 10 cells/ml PBS/0.1% BSA for OCM-1 and SP6.5 but not 92.1. Capture of cells at a concentration of 10 cells/ml PBS/0.1% BSA is comparable to the reported sensitivity of nested PCR observed in the literature [13]. Pre-labeling of Dyanbeads with only NKI/C3 failed to capture 92.1 at a concentration lower than 1x10^2^ cells/ml PBS/0.1% BSA.

In an attempt to improve the efficiency of immunomagnetic isolation of uveal melanoma cells, Dynabeads were pre-labeled with both NKI/C3 and NKI/Beteb. This combination of antibodies enabled the capture of all six cell lines down to a concentration of 10 cells/ml PBS/0.1% BSA. Pre-labeling of Dynabeads with a combination NKI/C3, NKI/Beteb, and PAL-M2 together failed to increase the sensitivity of the assay and actually prevented the isolation of 92.1, OCM-1, and SP6.5 below a concentration of 1x10^3^ cells/ml PBS/0.1% BSA. This observation in combination with the observation that NKI/C3 used alone is insufficient to permit the capture of 92.1 (which showed low staining intensity in 92.1) at concentrations comparable to those observed using PCR resulted in the omission of PAL-M2 from future assays.

In all cases, cells at concentrations of 1x10^6^ to 1x10^4^ cells/ml PBS/0.1% BSA were immediately discernable by light microscopy after performing the assay. Conversely, definitive identification of viable uveal melanoma cells at concentrations of 1x10^3^ to 10 cells/ml PBS/0.1% BSA was only possible after approximately five days of incubation at 37 °C in a humidified 5% CO_2_-enriched atmosphere.

### Isolation of uveal melanoma cells from spiked human blood

**Figure 2:**
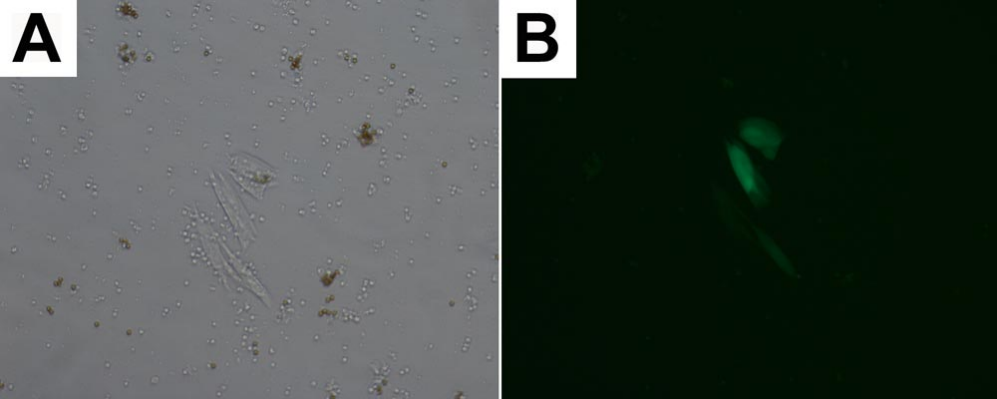
View of 92.1 cells isolated from spiked human blood. **A**: View at 10x magnification using conventional light microscopy of a colony of 92.1 transfected with GFP isolated from a sample of spiked human blood at a concentration of 10 cells/ml. **B**: View at 10x magnification using phase contrast microscopy of the colony of 92.1 transfected with GFP depicted in **A** isolated from a sample of spiked human blood at a concentration of 10 cells/ml.

Subsequent to validation and optimization of the immunomagnetic isolation method in culture, samples of blood from healthy volunteers were spiked with 92.1, MKT-BR, OCM-1, SP6.5, UW-1, or 92.1 transfected with GFP. Initially, blood samples were spiked with 92.1 transfected with GFP. This permitted easy identification of melanoma cells and their discrimination from other cell types present in the MNC layer as depicted in Figure 2A,B. In addition, this enabled us to identify a time frame in which we could expect to detect growth of melanoma cells at different concentrations. Immunomagnetic isolation from washed MNC layers using Dynabeads pre-labeled with monoclonal antibodies, NKI/Beteb and NKI/C3, permitted the capture of all five cell lines from a concentration range of 1x10^6^ to just 10 cells/ml human blood.

Immunomagnetic isolation of cell lines, 92.1, MKT-BR, SP6.5, and 92.1 transfected with GFP, from washed MNC layers using Dynabeads pre-labeled with monoclonal antibody 9.2.27 permitted the capture of 92.1, MKT-BR, and 92.1 transfected with GFP from a concentration of 1x10^6^ to 10 cells/ml human blood. SP6.5 could not be captured at a concentration below 1x10^6^ cells/ml human blood.

Cells at concentrations of 1x10^6^ to 1x10^5^ cells/ml human blood were immediately detectable by light microscopy after performing the isolation assay. Conversely, cells at concentrations of 1x10^4^ cells/ml human blood were only detectable by light microscopy within five to seven days of incubation at 37 °C in a humidified 5% CO_2_-enriched atmosphere.

## Discussion

The observation that nearly all uveal melanoma patients will test positive for the presence of CMCs over the course of their disease challenges the traditional concept of tumor progression. It would stand to reason that since only 50% of UM patients will die from metastasis, only a subset of patients would test positive for CMCs and that this would serve as a predictor for the development of metastases at a later date [1]. This does not appear to be the case. Thus, CMC positivity in itself is not an inherent predictor of metastasis in uveal melanoma and in itself, not of prognostic value. Studies in which immunomagnetic isolation of CMCs in cutaneous and uveal melanoma was used to quantify the presence of CMCs in blood and bone marrow concluded that the number of CMCs detected could be used to predict patient outcome [13,18]. In addition, some inherent properties of the CMCs themselves may offer insight into the propensity of a particular cell to form metastases, eventually leading to patient mortality.

Emerging evidence has challenged the notion that metastatic ability is a product of mutation and selection, which occurs in the late stages of tumorigenesis. Rather, the mutations associated with the emergence of a particular neoplasm confer its potential for metastasis with metastatic capability acquired early on in development [19,20]. Evidence supporting this hypothesis is abundant. In breast cancer, genetic signatures of poor prognosis observed in the primary tumor are highly predictive of patient outcome. In addition, studies have demonstrated the maintenance of global expression profiles between primary tumors and distant metastases [21].

Immunomagnetic isolation of CMCs in patients with cutaneous or uveal melanoma has revealed clonal relatedness between isolated cells with a population of cells demonstrating genetic profiles associated with poor prognosis. For example, patients with poor prognosis also had UM cells that exhibited monosomy of chromosome 3 [13]. Collectively, these results suggest that distinct populations of cells may have a predetermined capability to give rise to metastasis and that this potential can be identified before seeding of the metastatic site. The cell lines employed in this study all have known metastatic potentials as demonstrated in an animal model of uveal melanoma (UM) [22]. The detection of poor prognosis expression profiles in CMCs could be very useful in identifying patients at risk for the development of metastasis.

Further evidence suggests that in addition to a poor prognostic profile, tumors capable of metastasis possess sub-populations of cells, which exhibit tissue specific metastatic gene expression profiles [20]. Kang et al. [23] demonstrated the existence of distinct metastases gene expression profiles present in breast cancer cells that metastasize to the bone and of those which metastasize to the adrenal medulla. Cell populations shown to be highly metastatic to the bone were enriched in vivo, and it was found that the metastatic behavior was not associated with an increased expression of poor prognosis genes but with the upregulation of specific metastasis gene-expression profiles comprising of cell surface or secreted proteins. Similarly, Kakiuchi et al. [24] identified candidate genes that may be involved in organ specific metastasis in small cell lung cancer. Thus, in addition to genes indicative of poor prognosis, a distinct subset of genes exist which are related to the specifics of metastasis [23].

Initial data from this laboratory has similarly demonstrated changes in expression profiles of UM cells as they progress from the environment of the eye through the blood and the liver in an animal model of uveal melanoma. If identified in uveal melanoma, such genes could be useful in the classification of patients at risk for the development of metastasis as well as produce potential therapeutic targets. Immunomagnetic isolation of CMCs could therefore be extremely useful in the enrichment of such cell populations from patients.

Immunomagnetic isolation of CMCs from patients with various malignancies including colorectal cancer, breast carcinoma, osteosarcoma, cutaneous, and uveal melanoma has been demonstrated at sensitivities comparable to PCR [9,11-13,18]. However, in the latter case, the isolation of CMCs is performed using only the monoclonal antibody 9.2.27 [13]. Immunohistochemical profiling of our uveal melanoma cell lines revealed that the expression of a given cell surface protein is not necessarily consistent between different cell lines. Thus, an observation of high staining intensity with a given antibody on a particular cell line does not suggest an identical result in a different cell line. Our results therefore argue against the use of a single antibody for widespread application of CMC immunomagnetic isolation from uveal melanoma patients. Results clearly indicated that isolation of 92.1 using only NKI/C3 was inefficient and that to approach physiologically relevant sensitivity, a combination of NKI/C3 and NKI/Beteb had to be used. This combination of antibodies was found to be sufficient to permit the isolation of all cell lines tested at concentrations down to 10 cells/ml human blood. This result is significant as it has been postulated that disseminated tumor cells in patients without clinical metastases are present at very low frequencies (10–5-10–6) in bone marrow and lymph node [25].

The method for the isolation of circulating, cutaneous, and uveal melanoma cells outlined in the literature uses beads labeled with the 9.2.27 antibody alone [8,18]. Our results demonstrated that 9.2.27 used alone is insufficient to capture SP6.5 below a concentration of 1x10^6^ cells/ml whole human blood. Thus, use of this antibody alone may result in certain populations of circulating cells escaping detection. The question as to which combination of antibodies represents an optimum efficiency for the capture of CMCs remains unresolved. However, labeling with three antibodies does not appear to be more beneficial than labeling with two. In fact, our results suggest that it may actually hinder the isolation efficiency. The use of three antibodies failed to isolate 92.1, OCM-1, and SP6.5 at concentrations below 1x10^3^ cells/ml PBS/0.1% BSA

To the best of our knowledge, this is the first study to assess the efficiency of a panel of antibodies in their ability to permit immunomagnetic isolation of CMCs in uveal melanoma and the first study in North America to attempt to immunomagnetically isolate UM cells from blood. The initial results presented suggest that this method can be used to isolate CMCs at physiologically relevant concentrations and at sensitivities comparable to those seen in nested PCR. In addition, our data suggests that our method is more efficient and reliable for the isolation of CMCs in uveal melanoma than those methods currently in use. In addition, CMC isolation in an established animal model of uveal melanoma will permit further validation of this technique, providing a strong indication of the value of this method and its potential for use in a clinical setting.
